# The IL-1 receptor antagonist anakinra (kineret®) stabilizes the NLRP3 mutation-specific risk for hearing loss in patients with severe cryopyrin-associated periodic syndromes (CAPS)

**DOI:** 10.1186/1546-0096-12-S1-P76

**Published:** 2014-09-17

**Authors:** Mika Leinonen, Bengt Hallén, Hans Olivecrona

**Affiliations:** 1 Swedish Orphan Biovitrum, Stockholm, Sweden

## Introduction

CAPS is a rare monogenic autoinflammatory syndrome consisting of a spectrum of three conditions: Familial cold autoinflammatory syndrome (FCAS), Muckle-Wells syndrome (MWS), and the most severe form, NOMID/CINCA. Progressive hearing loss is a characteristic of severe CAPS [1]. Previous analyses showed that long-term anakinra treatment stabilized the progression of hearing loss [2,3]. CAPS patients with different NLRP3 mutations have distinctly different trajectories of hearing loss, suggesting a mutation-specific risk that should be considered when making treatment decisions [4].

## Objectives

To characterize the correlation between different NLRP3 mutations and hearing loss in severe CAPS patients and to evaluate whether long-term anakinra treatment stabilizes the progression of hearing loss.

## Methods

A prospective open-label study with long-term extension including 43 patients with severe CAPS was conducted at the National Institutes of Health [1,2]. The patients were treated with anakinra for up to 5 years. The patients who presented with NLRP3 mutations at baseline were further classified based on the gene location (D303N versus other locations). Hearing was monitored with audiogram which was evaluated with four frequency (0.5/1/2/4 kHz) pure tone average (4F-PTA), based on both air and bone conduction in the ear with best and worst hearing. The longitudinal changes in 4F-PTA were estimated with a mixed model for repeated measures (MMRM).

## Results

Altogether 22 patients provided pre and post treatment audiogram data. NLRP3 mutation was identified in 18 (82%) patients, 7 (32%) presented with D303N and 11 (50%) with other locations (2 x T348M, G569R, L264F, A374N, F443L, G326E, L632F, Q600P, V262A, V351L). In a multivariate analysis, the baseline 4F-PTA (ear with best hearing, air conduction) correlated with both age (p=0.032) and location of NLRP3 mutation (p=0.049) so that older patients and patients with mutations outside of D303N presented with more hearing loss. Following the initiation of the anakinra treatment, no significant changes were seen in mean 4F-PTA at any time point up to 5 years in either patients with D303N or patients with other NLRP3 mutations in the MMRM analysis adjusting for age; thus, hearing remained stable. Comparable findings were seen in the worst ear based on air conduction and for best/worst ear based on bone conduction assessments.

## Conclusion

Mutation-specific risk for hearing loss which is independent of age was seen based on the baseline audiogram data. Anakinra treatment for up to 5 years stabilized the progression of hearing loss regardless of the mutation.

## Trial registration identifying number

NCT00069329

## Disclosure of interest

M. Leinonen Consultant for: Swedish Orphan Biovitrum, B. Hallén Employee of: Swedish Orphan Biovitrum, H. Olivecrona Employee of: Swedish Orphan Biovitrum.
